# Supplementary material for: Epigallocatechin-3-Gallate Improves Intestinal Gut Microbiota Homeostasis and Ameliorates Clostridioides difficile Infection
Source: Nutrients. 2022 Sep 11;14(18):3756. doi: 10.3390/nu14183756 (PMC9504111; doi:10.3390/nu14183756)

Epigallocatechin-3-gallate improves intestinal gut microbiota homeostasis and ameliorates *Clostridioides difficile* infection

Supplementary Methods

16S rRNA gene sequencing

Fecal DNA was collected from mice prior to execution and isolated using the DNA PowerSoil Kit (Qiagen, Valencia, USA). The V3-V4 variable region was amplified using universal primers (Table S1). The PCR products were extracted, purified and sequenced on an Illumina NovaSeq platform. Raw sequencing datas were filtering, denoised, merged and detect using DADA2 [1] with QIIME2 [2]. The representative reads of amplicon sequence variants (ASVs) were annotated and blasted against Silva database Version 138. The microbial diversity was estimated using the alpha diversity (Shannon, Simpson, and Chao1 indices). Beta diversity Principal coordinates analysis (PCoA) was performed based on Bray–Curtis. Linear discriminant analysis (LDA) coupled with effect size measurements (LEfSe) was used to compare composition differences between groups. The 16S rRNA gene sequencing data are available at NCBI's Sequence Read Archive (SRA) database under the BioProject accession code PRJNA867379.

References

1. Callahan, B.J.; McMurdie, P.J.; Rosen, M.J.; Han, A.W.; Johnson, A.J.; Holmes, S.P. DADA2: High-resolution sample inference from Illumina amplicon data. Nat Methods 2016, 13, 581-583, doi:10.1038/nmeth.3869.

2. Bolyen, E.; Rideout, J.R.; Dillon, M.R.; Bokulich, N.A.; Abnet, C.C.; Al-Ghalith, G.A.; Alexander, H.; Alm, E.J.; Arumugam, M.; Asnicar, F.; et al. Reproducible, interactive, scalable and extensible microbiome data science using QIIME 2. Nat Biotechnol 2019, 37, 852-857, doi:10.1038/s41587-019-0209-9.

Supplementary Table S1. PCR primers.

Gene	Forward Sequence (5'-3')	Reverse Sequence (5'-3')
β-actin	AGTGTGACGTTGACATCCGT	GCAGCTCAGTAACAGTCCGC
Occludin	TTCCTCTGACCTTGAGTGTGG	CTCTTGCCCTTTCCTGCTTT
ZO-1	GCCGCTAAGAGCACAGCAA	GCCCTCCTTTTAACACATCAGA
MUC2	ATGCCCACCTCCTCAAAGAC	GTAGTTTCCGTTGGAACAGTGAA
Bacteria16S rRNA V3V4	TACGGRAGGCAGCAG	AGGGTATCTAATCCT

Supplementary Figure S1. The rarefication curve of ASVs.

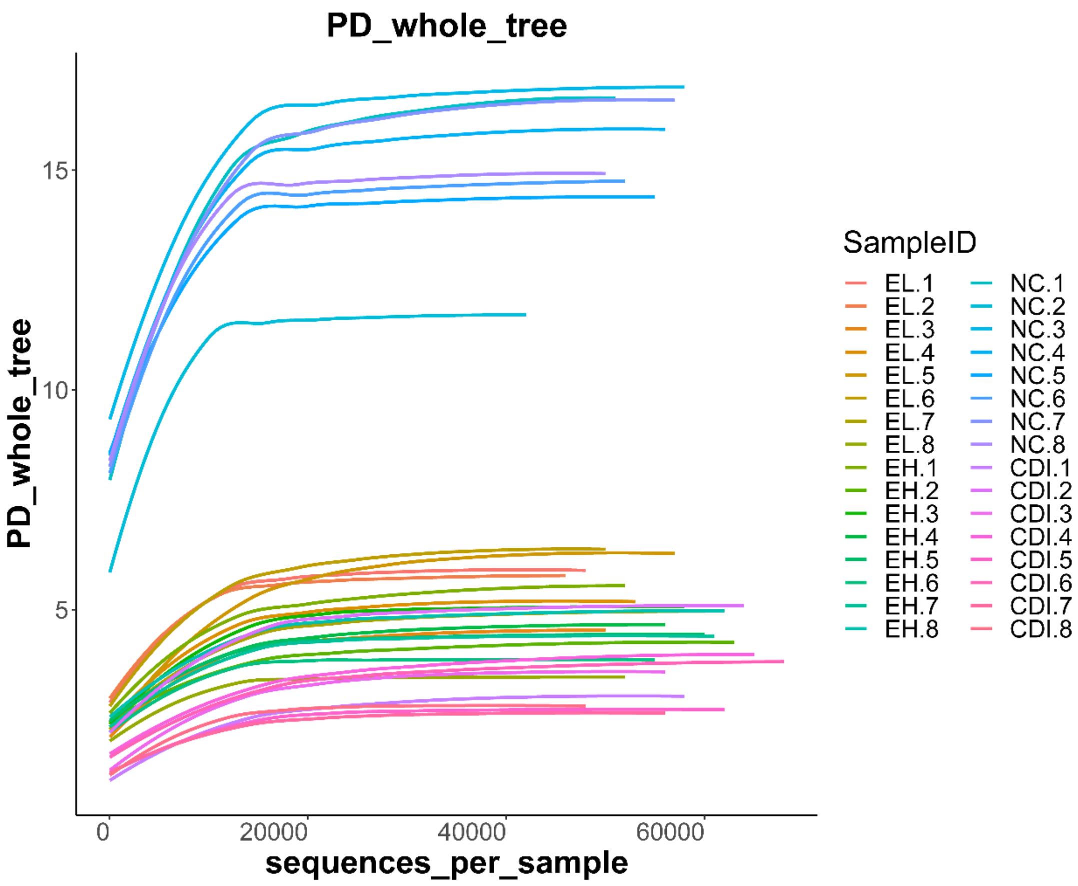

Supplementary Figure S2. Relative abundance of the most abundant taxa at the genus level.

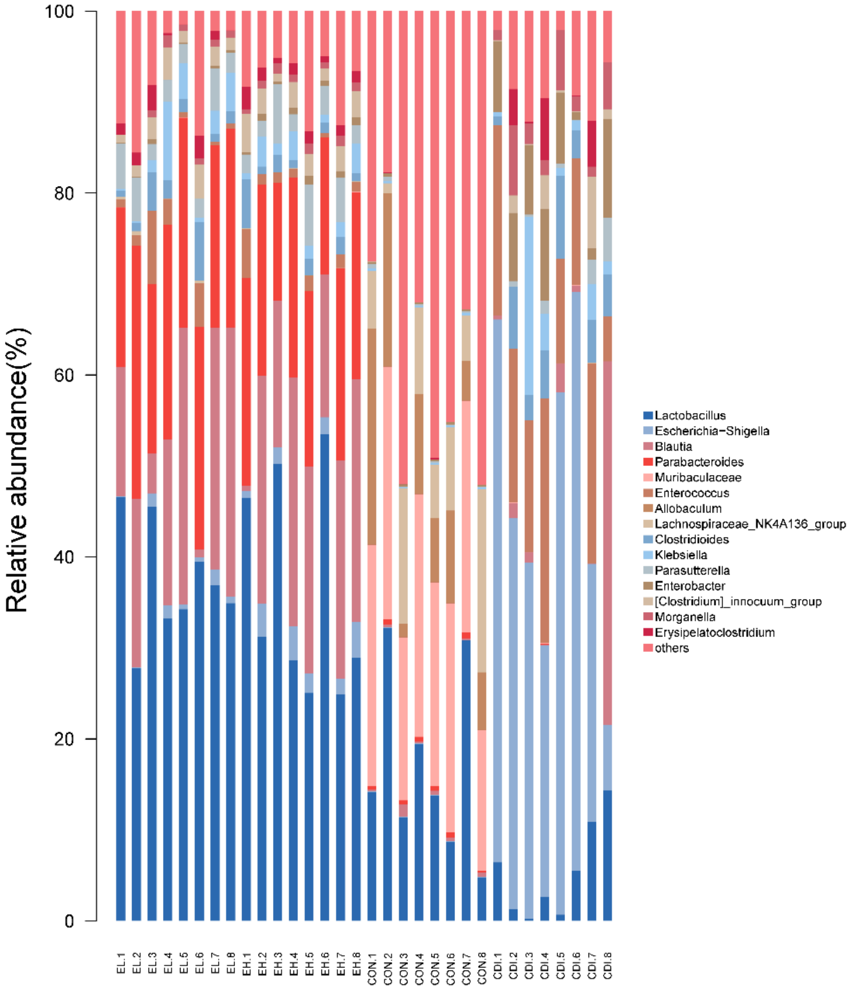

Supplementary Figure S3. Top ten abundance of differential taxa between groups.

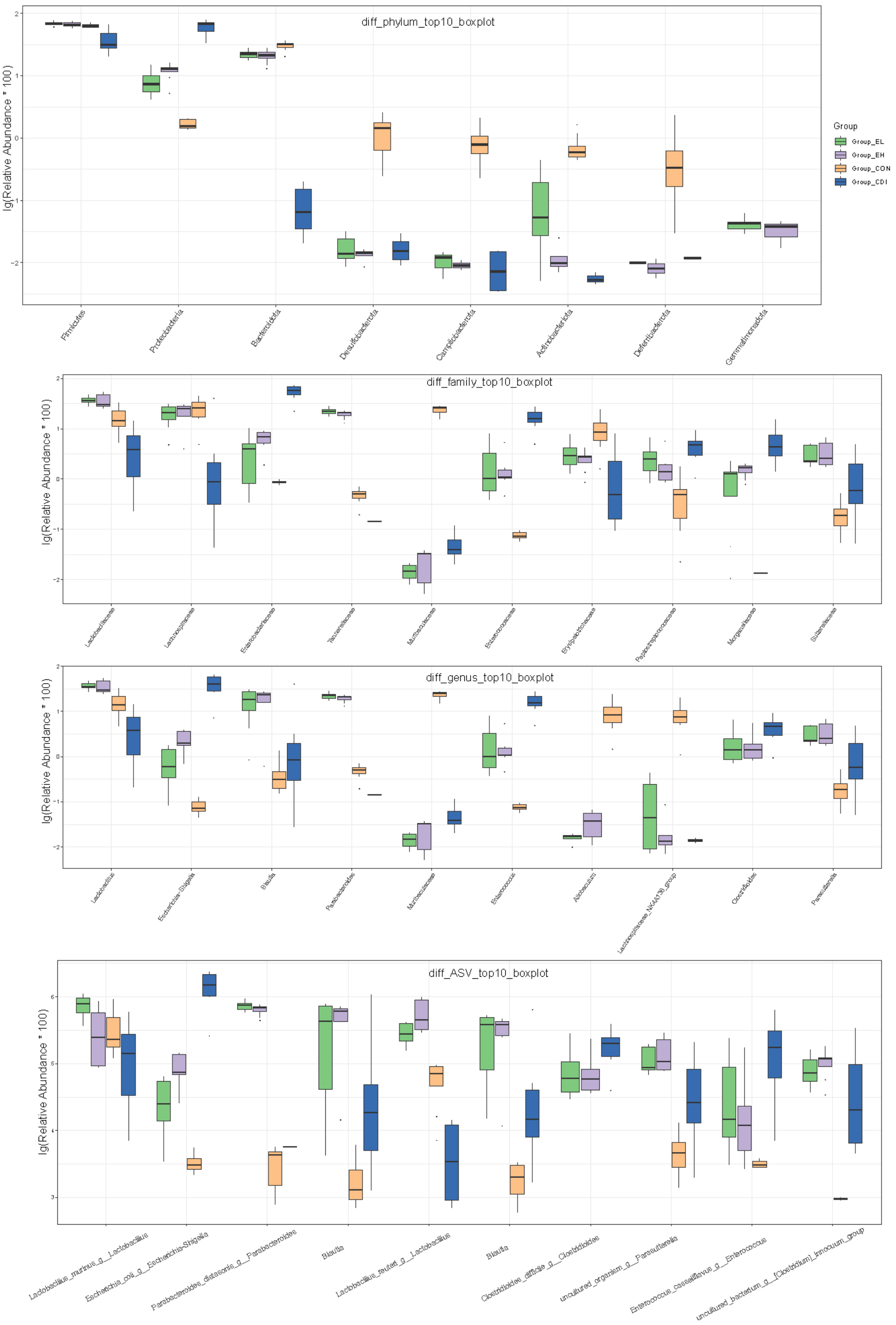

Supplementary Figure S4. Linear discriminant analysis (LDA) effectsize (LEfSe) analysis among CON and CDI group.

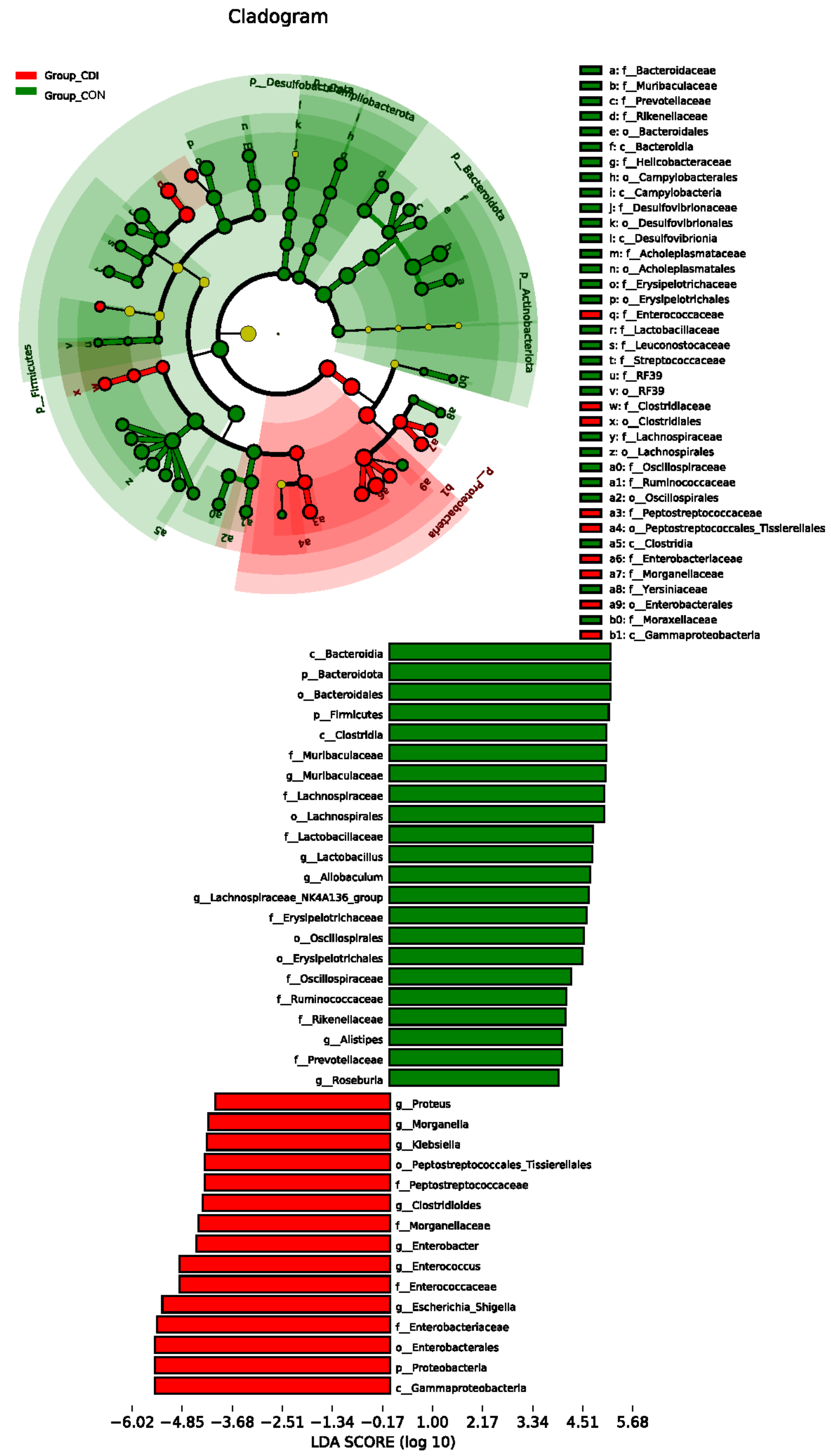

Supplementary Figure S5. The bacterial taxa of the EH group were compared with those of the CDI group.

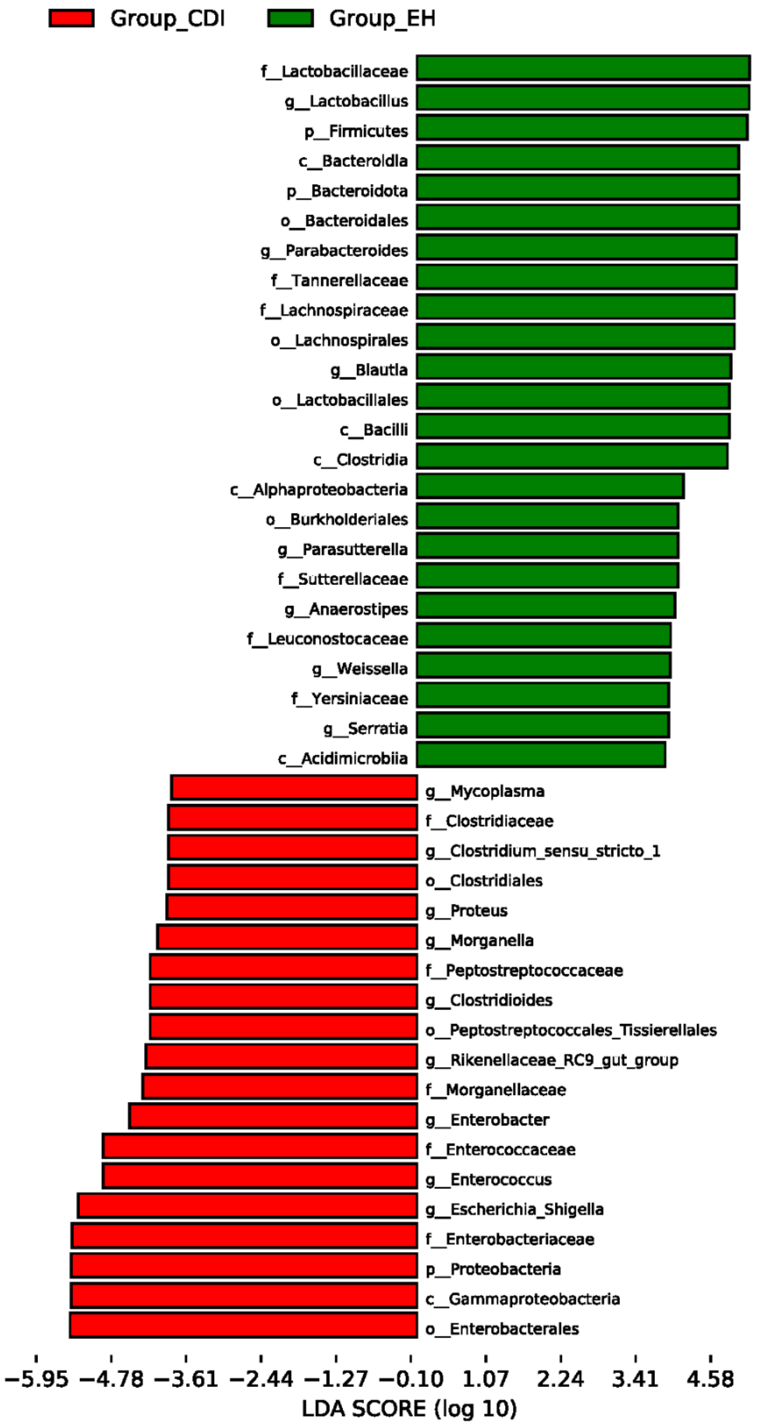

Supplementary Figure S6. Linear discriminant analysis (LDA) effectsize (LEfSe) analysis among EL and CDI group.

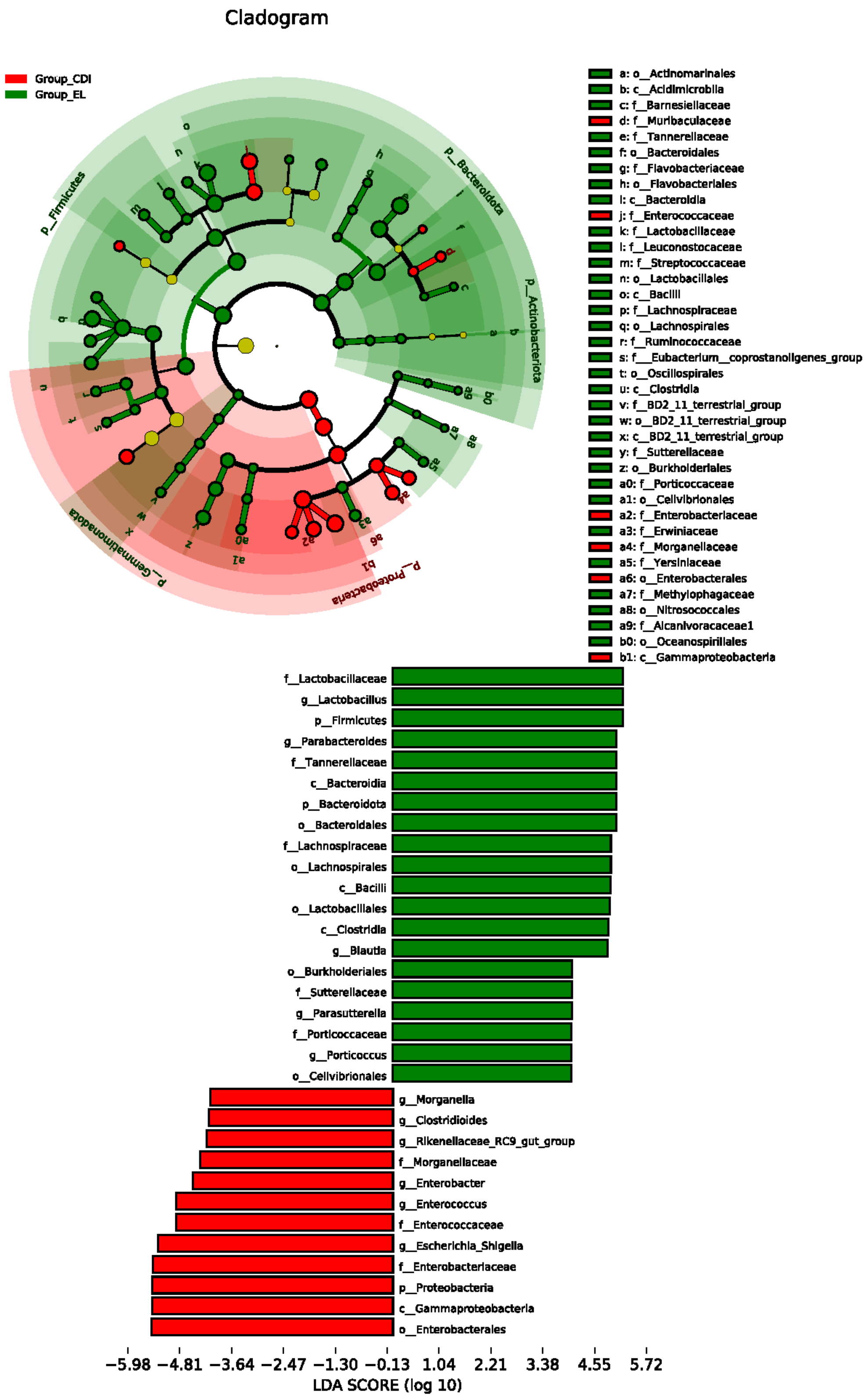

Supplementary Figure S7. OPLS-DA plot showing the metabolic profiles of the CDI and EL groups.

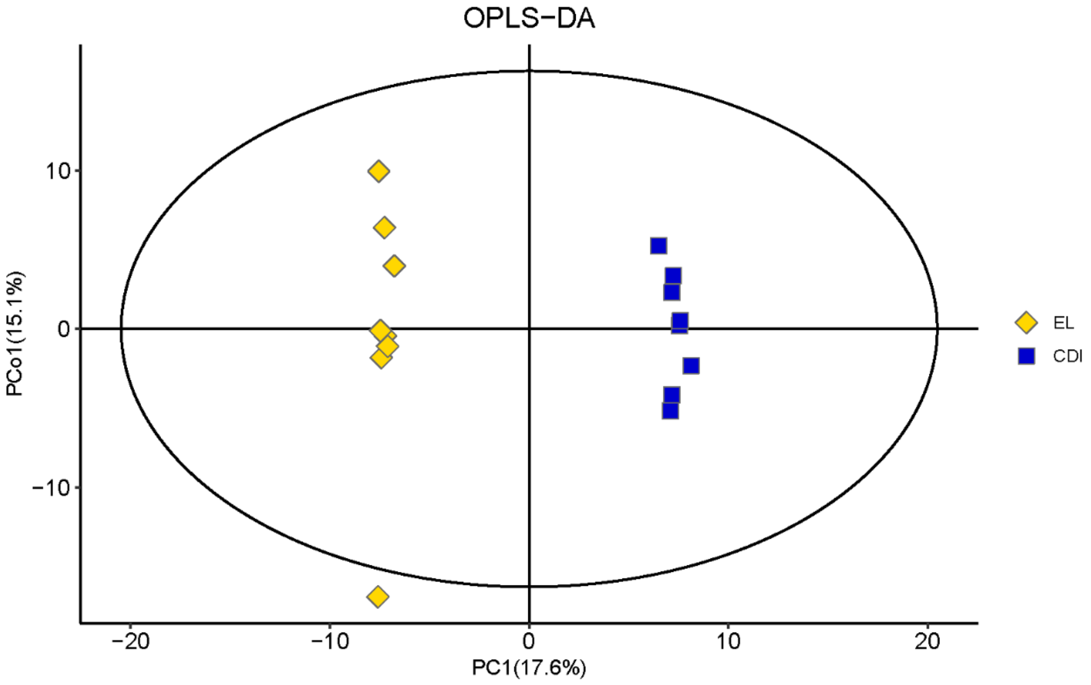

Supplementary Figure S8. A hierarchical clustering heatmap of normalized (Z-score) levels visualizing significantly altered metabolites between the CDI and EH groups.

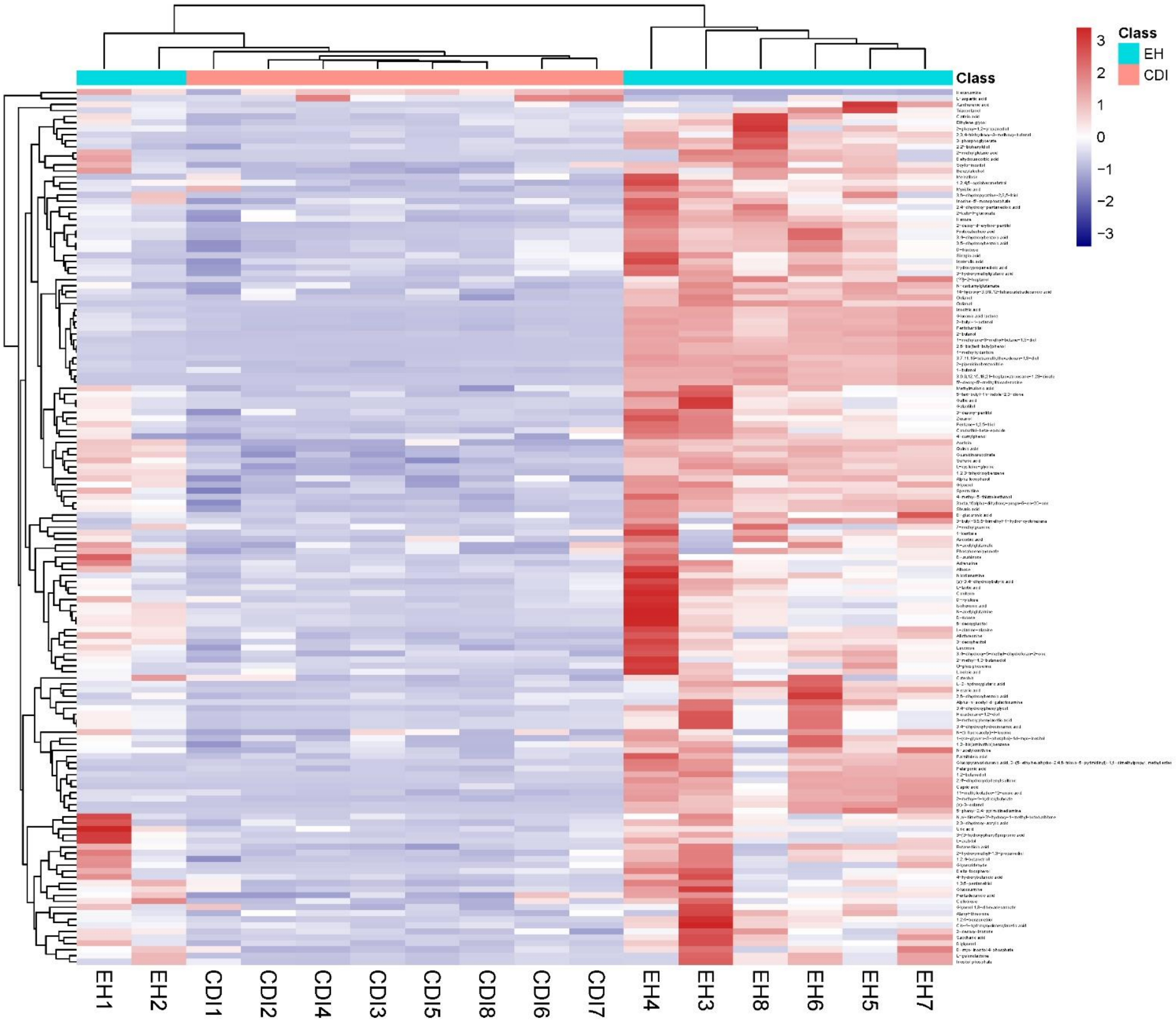

Supplementary Figure S9. The levels of SCFAs between different groups. The data are expressed as the mean ± SEM. *, $P < 0.05$; *, $P < 0.01$; and **, $P < 0.001$.

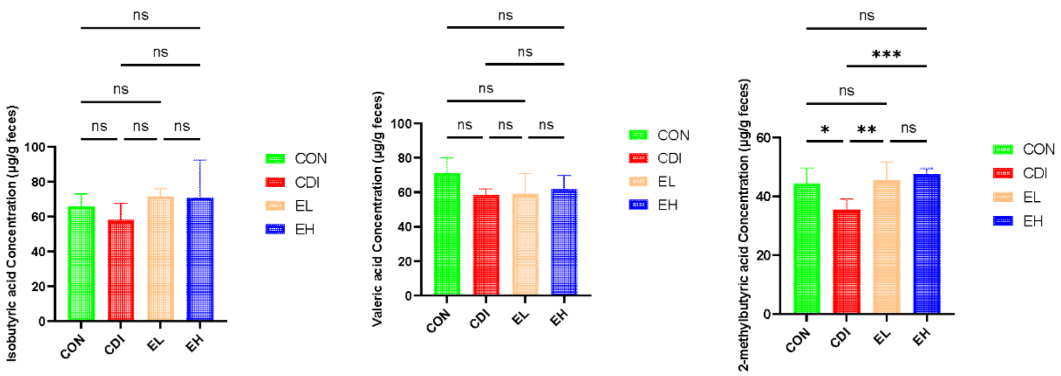

Supplement: Supplementary file 1 [file nutrients-14-03756-s001.zip › nutrients-1882848-supplementary.pdf]
